# Magnitude and the underlying reasons for routine prophylactic antibiotic use after uncomplicated vaginal births: A mixed-methods study in Eastern Uganda

**DOI:** 10.1136/bmjph-2025-004168

**Published:** 2026-07-21

**Authors:** Joshua Epuitai, Rose Chalo Nabirye, Ivan Lume, Proscovia Nabachenje, Sarah Racheal Akello, Turyasiima Munanura, Brenda Nabawanuka, Jacob Stanley Iramiot

**Affiliations:** 1 Busitema University, Tororo, Uganda; 2 Department of Nursing and Midwifery, Busitema University, Tororo, Uganda; 3 Department of Physiology, Busitema University, Tororo, Uganda; 4 Department of Pediatrics and Child Health, Busitema University, Tororo, Uganda; 5 Muni University, Arua, Uganda; 6 Department of Pediatrics and Child Health, Kampala International University, Kampala, Uganda; 7 Department of Nursing and Midwifery, Mountains of the Moon University, Fort Portal, Uganda

**Keywords:** Public Health, Cross-Sectional Studies, Qualitative Research

## Abstract

**Introduction:**

Routine prophylactic antibiotic use is not recommended following uncomplicated vaginal births because of the global threat of antimicrobial resistance (AMR). The study was conducted to determine the magnitude and underlying reasons for routine antibiotic use following uncomplicated vaginal births.

**Methods:**

We conducted an explanatory sequential study among women with uncomplicated vaginal births. We interviewed 370 women following facility discharge using interviewer-administered questionnaires. Eight in-depth interviews were conducted among midwives and clinical officers. Descriptive statistics and thematic analysis were used to analyse the data. The study obtained ethical clearance.

**Results:**

The median age of the participants was 25 (interquartile range 20–30). Out of the 370 women, 34% (n=127/370) had perineal tears, 19% (n=69/370) had preterm prelabour rupture of membranes and 6% (n=23/370) had preterm labour. Almost all (99%) women with uncomplicated vaginal births were prescribed prophylactic antibiotics during facility discharge. A combination of amoxicillin and metronidazole (49%, n=181/366) and ampiclox and metronidazole (40%, n=147/366) were the commonly prescribed drugs during discharge. Antibiotics were routinely given to prevent puerperal infections. Perineal tears, episiotomies and unsterile environment were additional justification given for antibiotic use. Sterility was seen to be untenable which necessitated antibiotic use to cover for gaps in maintaining infection prevention and control measures. In some cases, antibiotics were considered necessary regardless of the absence of medical indications and the risk of AMR. Healthcare providers had inadequate awareness of the risks of AMR and the guidelines on antibiotic use during discharge.

**Conclusions:**

Widespread routine antibiotic use after uncomplicated vaginal births reflected practices of overmedicalisation of childbirth and limited awareness of AMR. Developing local policies, strengthening infection prevention and control measures and innovating antimicrobial stewardship initiatives may be critical in reducing unnecessary antibiotic use after delivery.

**WHAT IS ALREADY KNOWN ON THIS TOPIC**

- Routine antibiotic use after uncomplicated births is not recommended because of the associated risk of antimicrobial resistance.
- Routine antibiotic use after uncomplicated births is common.

**WHAT THIS STUDY ADDS**

- Our study underscored almost universal antibiotic prescriptions after uncomplicated vaginal births.
- This was attributed to the need to prevent puerperal sepsis. Prophylaxis was deemed necessary following perineal tears, and gaps in maintainence of sterility. Routine antibiotic use occured from limited awareness of guidelines on antibiotic use and failure to attribute their prescription of antibiotics to antimicrobrial resistance.

**HOW THIS STUDY MIGHT AFFECT RESEARCH, PRACTICE OR POLICY**

- Local settings should develop clear guidelines on antibiotic use after uncomplicated vaginal births.
- Training and mentorship of healthcare workers is needed to promote rational antibiotic use after uncomplicated vaginal births.

## Introduction

 Puerperal sepsis is a genital tract infection that occurs between the onset of labour and the 42nd day postpartum.^1^ Puerperal sepsis is characterised by two or more clinical signs, which include fever, foul vaginal discharge, pelvic pain and delayed uterine involution.^1^ Globally, puerperal sepsis accounts for 11% of maternal mortality.^2^ In Uganda, puerperal sepsis causes approximately 30% of maternal deaths.^3^ Beyond mortality, puerperal sepsis causes considerable maternal near miss, including disability from secondary infertility and chronic pelvic pain.^4,5^ Consequently, prophylactic antibiotics are recommended to prevent puerperal sepsis in conditions that pose a substantial risk of puerperal sepsis.^1^ Conditions with significant risk of puerperal sepsis include caesarean births, third and fourth degree perineal tears, preterm prelabour rupture of membranes of less than 36 weeks gestation, manual removal of placenta, chronic conditions (eg, diabetes), bacterial vaginosis and group B streptococcus colonisation.^1,6^

However, antibiotics are considered unnecessary in uncomplicated vaginal births, defined herein as vaginal births without any clinical signs of infection or risk factors for postpartum infection.^1^ In addition, the World Health Organisation (WHO) does not recommend antibiotic use following preterm labour with intact membranes, meconium staining, operative vaginal delivery, episiotomy and second-degree tears.^1^ Following uncomplicated vaginal births, 98% of women are unlikely to develop puerperal sepsis.^1^ Consequently, strong evidence underscores that antibiotic use does not confer any clinical benefit in reducing the risk of puerperal sepsis in uncomplicated vaginal births.^1^ Therefore, the WHO strongly recommends against antibiotic use for uncomplicated vaginal births.^1^ The recommendation was based on the critical need to prevent a high rate of antibiotic misuse, which would pose a consequent public health implication of negating global efforts to reduce antimicrobial resistance (AMR).^1^

Globally, study findings have implicated a high burden of AMR in low- and middle-income countries.^7^ Studies in Uganda have cited remarkable AMR to commonly prescribed antibiotics.^8,9^ In Western Uganda, 82% of the isolated organisms exhibited multidrug resistance to the drugs used for management of puerperal sepsis.^8^ AMR is linked to poor health outcomes and prolonged hospital admissions.^8,10^ Globally, AMR was attributed to cause about five million deaths in 2019.^11^ In Africa, AMR was associated with more than one million mortalities in 2019.^11^ Misuse of antibiotics wastes finite antibiotic resources, and it increases healthcare expenditure.^10^ Antibiotic misuse may contribute to the epidemic of over-medicalisation of normal childbirth, where obstetric interventions are used without any clinical benefit and sometimes even have harmful health effects.^12^ Particularly, such harmful effects of misuse of antibiotics in puerperium include maternal anaphylaxis and development of neonatal allergies from disturbance of gastrointestinal normal flora.^1^

Despite the strong recommendation against antibiotics in uncomplicated births, there is widespread routine antibiotic use even in low-income countries.^6,13–15^ The WHO estimated that almost half (47%) of women with no medical indication received prophylactic antibiotics after low-risk childbirth.^6,13^ In Asian countries, antibiotic use following uncomplicated vaginal birth was nearly 100%.^16^ In Africa, antibiotics were used in 25% of women with uncomplicated births.^6^ Antibiotics were overused in Tanzania following a normal vaginal delivery, with the proportion ranging from 1.4% to 63%.^14^ Antibiotic use after facility discharge was 12% in the Democratic Republic of Congo, 6% in Kenya, and 8.5% in Zambia, which underlines significant use of antibiotics after delivery.^13^ Antibiotic misuse is disproportionately higher in the most deprived populations, where there is a tendency to self-medicate, hoard, and share medicines.^6,13^ The magnitude and the underlying factors of antibiotic misuse in uncomplicated births are not known in our setting. The study was conducted to determine the prevalence and the underlying reasons for routine antibiotic use among women with uncomplicated vaginal births.

## Methods and materials

### Study design and setting

We used an explanatory sequential mixed-methods approach where quantitative data were collected first, followed by qualitative data collection.^17^ Qualitative findings were used to enrich the quantitative findings through exploring the context and the underlying reasons for antibiotic use. The study was conducted among primary and tertiary health facilities in an urban setting in Eastern Uganda.

### Qualitative setting, sampling and study population

In the qualitative study, healthcare workers were recruited from two primary health facilities and one tertiary health facility. Primary health facilities were at the level of health centre III, which provided basic services such as uncomplicated vaginal births, and at the level of health centre IV, whose obstetric services extended to caesarean sections. The tertiary health facility was at the level of a regional referral hospital, which was manned by obstetricians and midwives. The tertiary health facility provided basic and emergency obstetric services, including caesarean sections. Purposive sampling was used to select healthcare providers. Purposive sampling and maximum variation were used to enable the selection of participants who could provide rich in-depth data. Healthcare workers who were working in labour suites, maternity/postnatal units for a longer time, and those heading these units were prioritised for selection. Healthcare workers were mainly midwives, nurses and clinical officers. Medical and clinical officers prescribe drugs after delivery, while in some cases, midwives may prescribe drugs, including antibiotics, after delivery.

### Qualitative data collection methods

Eight in-depth interviews were conducted in May 2025. The sample size was based on the context and practical implications of qualitative sampling.^18^ Participants were approached from their work stations and invited to join the study. All the participants who were approached accepted to join the study, except in cases where it was not possible to make an appointment with the study participants. Interviews were conducted in a private room, while non-participants were not allowed to be present during the interviews. Data collection was done by a final-year female nursing student who was in her early twenties. The interviewer was trained in qualitative data collection. Some of the participants might have known the researcher before data collection. An interview guide was developed to explore the underlying reasons for antibiotic use after uncomplicated births. The questions in the interview guide served to explore underlying perceptions, beliefs and reasons for the prescription of antibiotics following uncomplicated vaginal births. The interviews were audio-recorded and were conducted in English. The interviews lasted about 10 min.

### Study population for the quantitative study

The quantitative study was conducted in a tertiary hospital from February 2025 to May 2025. Participants were recruited from the postnatal unit of the hospital. The study population comprised postpartum women who had uncomplicated vaginal births. We operationalised the WHO definition of uncomplicated vaginal birth as one where women who would not be eligible to receive antibiotics after delivery and during discharge from the facility.^1^ These included women who were due for discharge according to hospital policies. Women with first- and second-degree tears, episiotomy, preterm birth with intact membranes, preterm prelabour rupture of membranes from 36 weeks of gestation, meconium staining and operative vaginal delivery were eligible for the study.^1^ However, operative vaginal delivery was not considered in the study as it was not done in the health facility. Women who had a caesarean section were excluded from the study. Consecutive sampling was used to select the participants. This involved selecting all the eligible participants until the sample size was reached. The sample size was calculated using Kish and Lesley’s formula.^19^ The sample size of 370 participants was based on the 47% prevalence of antibiotic use, which was reported in low- and middle-income countries.^6^

### Data collection tools and study variables for the quantitative study

Interviewer-administered questionnaires (online supplemental file 1) were used to interview participants. We developed the questionnaire alongside findings from the literature review. We mapped clinical variables that were associated with antibiotic use after delivery. The participants were interviewed when they were discharged from the hospital. All the women approached agreed to participate in the study. The questionnaire consisted of three sections: (1) sociodemographic characteristics, (2) obstetric characteristics and (3) antibiotic use following normal delivery. The outcome variable was the overall antibiotic use during discharge which was determined by checking hospital records and the discharge form. This involved reviewing all medications given to women during facility discharge. Antibiotic use before and during delivery was also assessed through asking women if they had used antibiotics during pregnancy and delivery. Self-reports were used to collect sociodemographic, antenatal and obstetric history. The independent variables included sociodemographic characteristics (age, tribe, religion, level of education, marital status, level of income); obstetric factors (parity, number of antenatal care (ANC) visits, timing of first ANC visits, use of antibiotics during pregnancy, admission during pregnancy) and intrapartum care factors (duration of labour, number of vaginal examinations, preterm prelabour rupture of membranes, amniotomy, meconium staining, foul smelling, perineal tears, episiotomy, obstructed or prolonged labour, HIV status, manual removal of placenta). Multiple responses were reported for women who had more than one obstetric condition.

### Quantitative and qualitative data analysis

Transcripts were transcribed verbatim, while Braun and Clarke’s six steps of thematic analysis were used to analyse qualitative data.^20^ JE and SRA conducted inductive thematic analysis. STATA V.15 was used to analyse the quantitative data. Frequencies and percentages were used to analyse categorical variables. Median and interquartile range (IQR) were used for continuous variables because the data were not normally distributed.

## Results

### Description of the study participants

The median age of the participants was 25 (20–30) (table 1). The majority (85%) of the participants had secondary education or below. The majority (42%) of the participants were peasants or housewives, while 31% of them had only one child.

**Table 1:** Description of the study participants

Variable	Frequency (n=370)	Percentage
Age in years (median and IQR) 25 (20–30)		
Marital status		
Single/separated	35	9.5
Married/cohabiting	335	90.5
Level of education		
No education/primary	123	33.2
Secondary	192	51.9
Tertiary	55	14.9
Occupation		
Peasant/housewife	157	42.4
Self-employed	128	34.6
Formal employment	34	9.2
Unemployed	51	13.8
Tribe		
Iteso	29	7.8
Mugishu	233	63.0
Muganda	23	6.2
Others	85	22.0
Religion		
Christian	234	63.2
Muslim	136	36.8
Daily average income (in Uganda Shillings)		
Less than 4000	30	8.1
4000–10 000	141	38.1
More than 10 000	199	53.8
Number of children		
1	115	31.1
2–4	196	53.0
5 or more	59	15.9

### Description of the obstetric characteristics

During pregnancy, 22% (n=83/370) of the participants were admitted to the hospital (table 2). Only 6% (n=23/370) of the participants had preterm birth, while 19% (n=69/370) noted prelabour rupture of membranes. The majority (68%, n=256/374) of the women had uneventful labour free from complications. About 34% (n=127/370) of women had a perineal tear after birth.

**Table 2:** Obstetric-related characteristics

Variable	Frequency (n=370)	Percentage
Number of ANC visits		
1–3	115	31.1
4–7	196	53.0
8 and more	59	15.9
Inpatient admission during pregnancy		
Yes	83	22.4
No	287	77.6
HIV status		
Negative	321	86.8
Positive	15	4.1
Not sure	34	9.2
Gestation age at delivery		
Preterm	23	6.2
Term	334	90.3
Post-term	13	3.5
Preterm prelabour rupture of membranes		
Yes	69	18.7
No	301	81.3
Spontaneous rupture of membranes		
Yes	293	79.2
No	77	20.8
Complications during labour (n=374)*		
None	256	68.4
Obstructed/prolonged labour	59	15.8
Hypertensive disorders of pregnancy	24	6.4
Postpartum haemorrhage	10	2.7
Others†	25	6.7
Perineal tear during delivery		
No	234	65.7
Yes	127	34.3

* Multiple responses (women with more than one condition).

† Others: twin birth, breech birth, retained placenta, birth asphyxia, epilepsy, perineal tear, preterm prolonged rupture of membranes and preterm birth.

ANC, antenatal care.

### Antibiotic use during pregnancy, labour and after normal births

**Figure 1:**
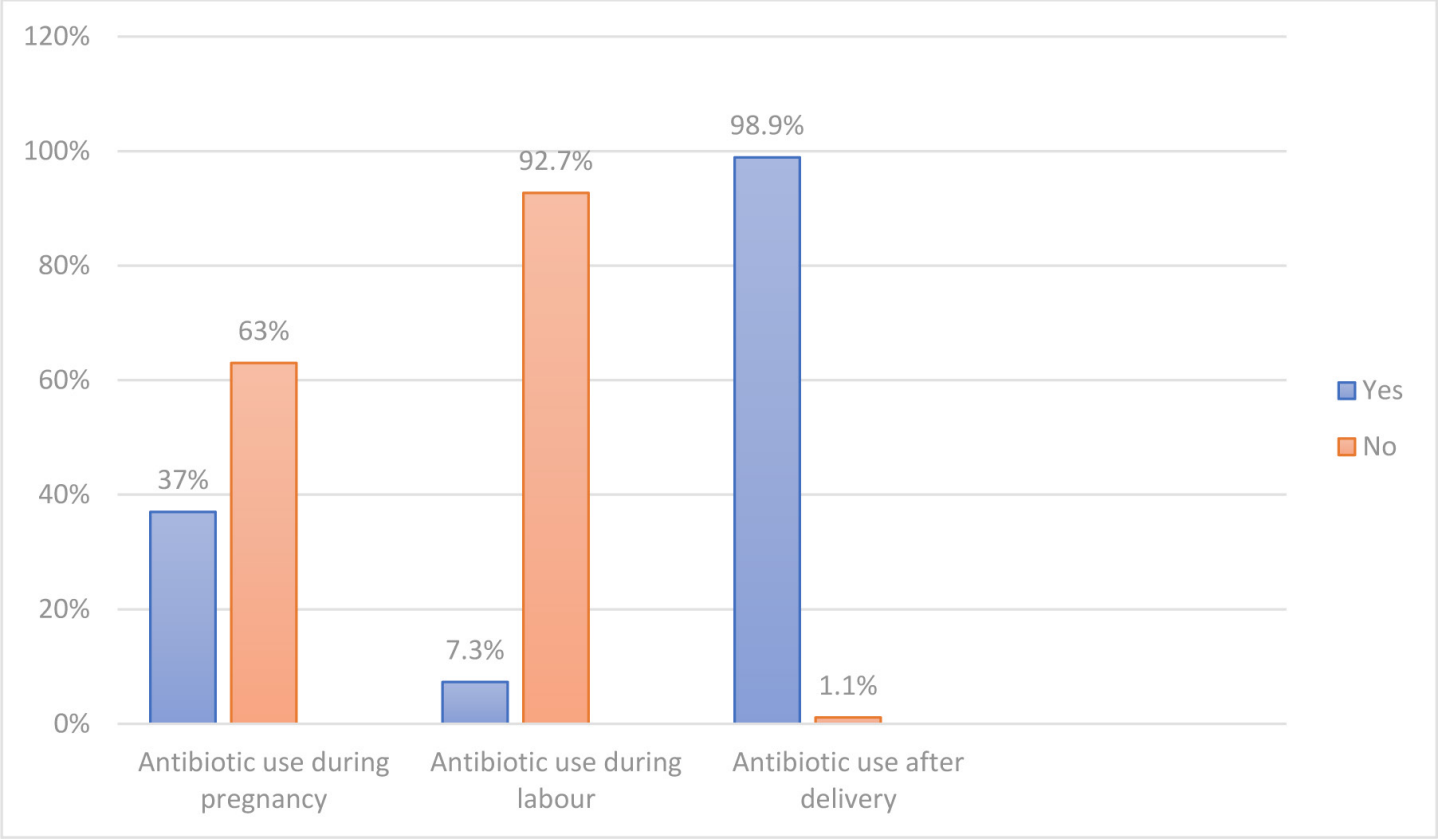
Antibiotic use during pregnancy, labour and after normal births.

Antibiotic exposure was frequent during pregnancy and after delivery (figure 1). More than one-third (37%) of women reported antibiotic use during pregnancy. Notably, nearly all participants (99%, n=366/370) were prescribed antibiotics after uncomplicated vaginal births during discharge.

### Types of antibiotics used after normal births

**Figure 2:**
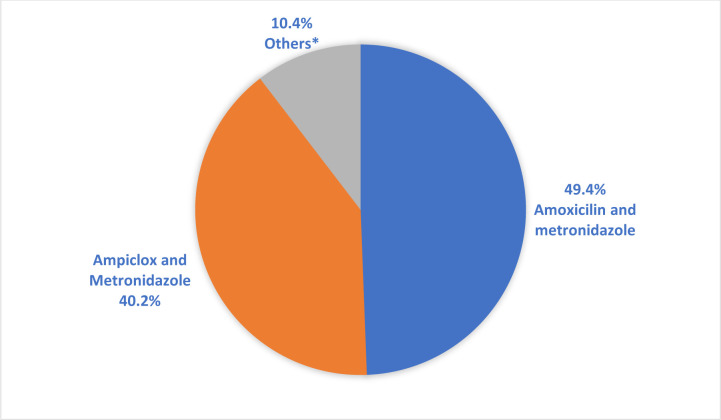
Antibiotics prescribed after normal births. *Others: Amoxicillin, Ampiclox, azithromycin, Flucamox and metronidazole.

The majority of prescriptions combined penicillin-type drugs with metronidazole (figure 2). Out of the 366 women who were prescribed antibiotics, 49% (n=181/366) were given amoxicillin and metronidazole during discharge. About 40% (n=147/366) were prescribed Ampiclox and metronidazole. The remaining 10% (n=38/366) received other single or combination regimens including amoxycillin, Ampiclox, azithromycin, metronidazole and Flucamox.

### Qualitative findings

#### Theme: Reasons for antibiotic use after uncomplicated births

Healthcare providers reported prescribing antibiotics for women after normal births. This was seen as generally a good practice for women who were due for discharge. The reasons given for antibiotic use were mostly to prevent puerperal infection, which was seen to occur from perineal tears, an unclean environment, vaginal and urinary tract infections. Limited knowledge regarding the effects of routine antibiotic use was seen to promote widespread use of antibiotics.

##### Subtheme: rophylaxis and treatment of infections

Participants used phrases such as ‘normally’ to indicate that antibiotic use after uncomplicated birth delivery was the norm and a routine practice. Antibiotics such as amoxicillin, ampicillin, ampiclox, ceftriaxone and metronidazole were routinely used. The choice of antibiotics was mostly based on their availability, affordability and perceived effectiveness. Antibiotics were given as prophylaxis against bacterial infections, puerperal sepsis, vaginal infections and complications after delivery.

For mothers who have had normal vaginal delivery,…, normally we use amoxycillin and metronidazole. (IDI 2)

I think it depends on the facility setting, on those facilities where you see someone…. if I write ampiclox®…that person may not afford ampiclox®. if the facilities have amoxyl [amoxycillin], then I give Amoxil[amoxycillin … There are facilities which are having all these things. And also, the capability of that very person. (IDI 1)

Women were considered to be at an increased risk of infection after delivery. This occurred from biological, social and cultural factors that predisposed women to infections after delivery. Women were considered to have low immunity, from a high vaginal pH, which favoured microbial growth. The low immunity was seen to justify antibiotic use after birth. Furthermore, exposure to infection was seen to occur from various sources, including toilets and washrooms. As such, antibiotic use was considered inevitable to prevent infection after normal vaginal delivery. The increased risk of infection and the importance of prevention left healthcare workers with no option but to prescribe antibiotics after uncomplicated deliveries.

They are exposed to surfaces and at the risk of developing infections, more so vaginal infections. That’s why we consider giving them antibiotics. (IDI 1)

Antibiotics were given as a preventive measure against urinary tract infections (UTIs). This was common for women with complaints and signs suggestive of UTIs, such as foul smell and abnormal vaginal discharge. However, investigations were not conducted to confirm diagnosis of suspected UTIs. In some cases, antibiotics were given as a form of treatment for women suspected of having UTIs.

Then also, you can tell when you’re delivering a mother, maybe there are discharges that are coming out of her, and a foul smell…then also, maybe she has been having UTI (IDI 4)

So, we want to give antibiotics….to prevent any infection if it has not yet been there, and if it’s there, to treat the infection. (IDI 1)

##### Subtheme: Antibiotics were given for perineal tears/episiotomies or for prolonged labour

Perineal tears and episiotomies were mentioned as a justification for prescribing antibiotics to women after uncomplicated births. Antibiotics were seen to promote healing and to prevent puerperal sepsis. Others identified prolonged labour as an indication for antibiotic use after birth.

I think antibiotics used in delivery is not bad because like in most areas….these mothers who get perineal tears, lacerations, you see that. For them to heal quickly, I think antibiotics are not bad (IDI 3)

While some identified obstetric conditions as reasons for providing antibiotics, others noted that antibiotic use was justifiable even for women with no medical indications. Some participants responded in the affirmative regarding the need to prescribe antibiotics even in cases where there was no medical indication. Others noted personal discretion and judgement to guide decision making to use antibiotics.

I think….any mother who has a vaginal delivery or even has no tear or episiotomy must be given an antibiotic (IDI 1)

##### Subtheme: difficulty in maintaining sterility

Women were seen to be exposed to infections from the frequent vaginal examinations (VEs) in addition to difficulty in maintaining sterility during VEs. This was fronted as a justification for antibiotic use after normal vaginal deliveries.

Remember, as we put our hands [inside to examine], we have an ascending infection….and our sterility. We find in some facilities; we don’t even sterilise……so….it is good you just give that [antibiotic] to the mother (IDI 1)

Even the frequent VEs, these mothers get during labor… you can’t really guarantee, though it’s done under aseptic measures, but you can’t really guarantee. (IDI 3)

Antibiotics were considered irrelevant in cases where sterility was maintained. However, in most of the facilities, sterility was considered difficult and unrealistic to maintain in the delivery room. Resource constraints, such as the unavailability of enough beds or personnel, meant that some women could deliver in unclean settings. Antibiotic use was seen as a good practice ‘just in case’ the woman was exposed to any infections during an uncomplicated delivery. The unsterile environment was seen to predispose women to infections, which, therefore, justified antibiotic use as a preventive measure against infections.

Yes, just in case…because I feel these mothers have been exposed. Like during delivery, there is a lot of exposure (IDI 5)

There is no need for antibiotics if you have observed your sterility, that may be 100%. In most facilities, we are not 100%….like in the case when… a mother delivers from down [on the floor]……like these mothers who deliver at from home. Do you know how she has done it? (IDI 3)

Besides the environment, women were also perceived to have poor hygiene and sanitary practices. This was common among women who delivered at home where it was difficult to guarantee their sterility following home deliveries. Antibiotic use was justified for women with no risk factors or medical indications because the healthcare workers were uncertain of the cleanliness and hygiene of the environment at home after discharge.

We don’t know if she’s going to eat. How she’s going to interact on the other side. So, I think it will be …better….if you prescribe some(IDI 6)

##### Subtheme: Limited knowledge of AMR

Healthcare workers were unaware of the guidelines regarding antibiotic use after uncomplicated vaginal births. When asked about the negative consequences associated with routine antibiotic use after delivery, some of the participants could not identify any associated adverse effects. Others noted AMR though they were uncertain whether routine antibiotic use would result in AMR. In some cases, participants maintained the need to prescribe antibiotics regardless of the likely risk of AMR.

The disadvantage I see about giving antibiotics is maybe the antibacterial resistance…yeah, that’s the major problem we are facing right now with most of the antibiotics. So, we give regardless (IDI 1)

## Discussion

The study was conducted to determine the magnitude of routine use of antibiotics following uncomplicated vaginal delivery. Overall, nearly (99%) of the women were prescribed antibiotics following normal vaginal births. Antibiotics were routinely given to prevent puerperal infections among women who were suspected of having vaginal infections, UTIs, perineal tears and prolonged labour. Besides, routine antibiotic use was linked to poor adherence to aseptic procedures, multiple VEs and the failure to maintain a sterile environment. Our study findings provide important implications for antimicrobial stewardship and the overmedicalisation of childbirth.

The WHO does not recommend routine use of antibiotics following uncomplicated vaginal births.^1^ Prohibiting antibiotic use would reduce the risk of AMR, especially given the minimal clinical benefits of routinely prescribing antibiotics in uncomplicated births.^1^ Nevertheless, our study findings underscored widespread antibiotic use, where 99% of women were prescribed antibiotics during discharge. Our findings were consistent with 99%–100% antibiotic use observed in Asia.^13,21^ Our study findings were higher than 10% antibiotic use noted among African sites from Kenya, Zambia and the Democratic Republic of Congo.^13^ The prevalent antibiotic use in our study setting reflected a routine practice where antibiotics were used regardless of the absence of medical indications. This suggested that the national and international guidelines that prohibit routine antibiotic use were not implemented in our setting.^22,23^ This could be related to providers’ level of awareness regarding the existence of guidelines on rational antibiotic use.^22,23^ In some settings, the existence of guidelines alone was not enough to influence judicious antibiotic use.^23,24^ In India, quality improvement efforts reduced routine antibiotic use to 10% through adapting international guidelines to the local setting, integrating evidence-based guidelines into routine practice, regular meetings and empowering stakeholders.^23^ Therefore, implementing such quality improvement interventions can effectively promote rational antibiotic use.^23,24^

In our setting, prophylaxis against infections and treatment of UTIs were the main reasons given for routine antibiotic use. Women were perceived to have weak immunity amidst pre-existing vaginal infections and UTIs. Training and empowering healthcare workers with information regarding the indications of antibiotic use during childbirth could address limited awareness and tendencies to routinely prescribe antibiotics. Although antibiotics were routinely given as prophylaxis in our setting, they are only indicated for prophylaxis against confirmed group B streptococcus.^1^ In our setting, laboratory investigations were not routinely conducted to diagnose suspected infections after childbirth. Instead, healthcare providers relied on clinical suspicion, a finding which has been observed in other settings.^24^ Relying on clinical suspicion rather than confirmed diagnosis is concerning, as it may promote unnecessary antibiotic use.^24^ Consistent with other study findings,^25^ sterility was considered unrealistic and untenable to achieve, given the resource constraints such as inadequate sterile equipment necessary for maintaining infection prevention and control in our setting. This resulted in gaps in the implementation of infection prevention and control practices.^25^ Routine antibiotics were provided to cover for lapses in the maintenance of sterility during childbirth. Antibiotic use occurred from providers’ fear of the adverse effects of infections and the need to prevent these infections.^22^ This zealousness to prevent infections could be channelled to promote efforts that strengthen infection prevention and control practices. This would include minimising the number of VEs, ensuring aseptic techniques and maintaining clean and sterile environments, which ultimately would reduce the need to prescribe antibiotics.^1,26^

Despite recent evidence that prophylactic antibiotics reduced the risk of perineal infections among women with first/second degree perineal tears, it was not of sufficient quality to warrant guideline change.^26^ As a result, the existing guidelines do not recommend prophylactic antibiotic use in episiotomies and first/second degree perineal tears.^1^ Nevertheless, in our setting, perineal tears and episiotomies were cited as one of the indications for antibiotic use. Almost all women were given antibiotics, which suggests that perineal tears could have been used as a cover-up for the widespread culture of irrational use of antibiotics after uncomplicated vaginal birth. Consistent with other findings,^25^ healthcare workers noted the need to provide antibiotics even in the absence of medical indications and regardless of the risk of AMR. Healthcare workers tend to have limited knowledge on AMR while some of them do not think their prescription of antibiotics can lead to AMR.^25^ The routine antibiotic use may be driven by institutional practices, absence of local guidelines, pressure from supervisors and limited knowledge regarding the impact of AMR.^22,23^ Promoting non-antibiotic interventions, such as maintaining sterile environments during perineal repairs and antimicrobial stewardship practices, can significantly reduce the risk of infection and the need to use antibiotics among women with less severe perineal tears.^1^

### Study strengths and limitations

The study reports antibiotic use from a resource-constrained setting. The widespread use of antibiotics meant that our study was not powered enough to determine the factors associated with the use of antibiotics following uncomplicated vaginal births. Nevertheless, the qualitative findings were used to provide a rich contextual understanding of the likely reasons for antibiotic use after discharge. We relied on self-reports to collect antenatal and intrapartum history. Recall bias and misclassification bias, especially for clinical variables, were likely in the study which could have affected the quality of the data collected. We checked the discharge form and hospital record book to determine the antibiotic use after delivery, which helped to reduce recall bias in the study.

## Conclusions

Routine antibiotic use following uncomplicated vaginal births was nearly universal in our study setting, despite strong WHO recommendations against this practice. Prescriptions were largely justified by concerns over puerperal sepsis, perineal tears and unsterile environments. However, these practices emanated from limited awareness of AMR, gaps in infection prevention and control and a culture of overmedicalisation of childbirth. Reducing unnecessary antibiotic use requires adapting international guidelines to the Ugandan context with clear recommendations on prophylactic antibiotic use after uncomplicated vaginal births. Engaging healthcare workers through training and mentorship is critical to strengthen infection prevention and control practices. This could reduce provider reliance on antibiotics as a compensatory measure, but it may also promote appropriate antimicrobial stewardship practices that safeguard against AMR. Ultimately, tackling this widespread misuse of antibiotics is critical not only for safeguarding maternal and newborn health in Uganda but also for contributing to the global fight against AMR.

## Supplementary material

10.1136/bmjph-2025-004168 online supplemental file 1

## Data Availability

Data are available on reasonable request. All data relevant to the study are included in the article or uploaded as supplementary information.
