# Supplementary material for: Magnitude and the underlying reasons for routine prophylactic antibiotic use after uncomplicated vaginal births: A mixed-methods study in Eastern Uganda
Source: BMJ Public Health. 2026 Jul 21;4(3):e004168. doi: 10.1136/bmjph-2025-004168 (PMC13404502; doi:10.1136/bmjph-2025-004168)
Supplement: online supplemental file 1 [file bmjph-4-3-s001.pdf]

Data collection tool-routine antibiotic use after delivery

Interviewer-administered Questionnaire

1. What is your age?...........................
2. What is your marital status?
    a. Married/cohabiting
    b. Single/separated
3. What is your level of education?
    a. No education/primary
    b. Secondary
    c. Tertiary
4. What is your occupation?
    a. Peasant/housewife
    b. Self-employed
    c. Formal employment
    d. Unemployed
5. What is your tribe?
    a. Iteso
    b. Mugishu
    c. Muganda
    d. Others (specify)…………………..
6. What is your religion?
    a. Christian
    b. Muslim
    c. Others (specify)………………….
7. How much do you spend on average in a day in UGX?
    a. <4000
    b. 4,001-10,000
    c. 10,000-50,000
    d. >50,001
8. How many children do you currently have?........................
9. How many pregnancies have you had so far?............................

10. Did you attend ANC during pregnancy?
    a. Yes
    b. No
11. If yes, how many ANC visits did you attend during pregnancy?.......
12. Were you admitted in the hospital during pregnancy?
    a. Yes
    b. No
13. If yes, how many times were you admitted in the hospital?................................
14. What is your HIV status?
    a. Positive
    b. Negative
15. What medicines were you given to take during pregnancy?
    a. None
    b. Iron-folate
    c. Penicillin
    d. Cephalosporins
    e. Others (specify)…………………………………………………………………………… ………………………………………………………………………………………… ……………………………………………………………………………
16. If yes, what antibiotics did you use?
    a. Penicillin
    b. Cephalosporins
    c. Macrolides
    d. Others (specify)……..
17. What was the gestation age of this baby?
    a. Preterm
    b. Term
    c. Post-term
18. How long did your labour last?...
19. Did your membranes rapture before term and before labour begun?

a. Yes
b. No

20. Did your membranes rapture spontaneously?
    a. Yes
    b. No
21. What was the colour of the liquor when the membranes raptured?
    a. Colourless
    b. Brownish-greenish
    c. Not sure
22. What was the duration between the rapture of membranes and the time when you delivered?..
23. What cadre conducted your delivery?
    a. Student
    b. Midwife/nurse
    c. Doctor
24. Did you have any complication during labour?
    a. Obstructed/prolonged labour
    b. Hypertensive disorders
    c. Twin delivery
    d. Others (specify)…………………………………………………..
25. Did you have a perineal tear during delivery?
    a. None
    b. First to second degree
    c. Third to fourth degree
    d. Episiotomy
26. What medicines were you given to take before coming to the hospital to delivery your baby?
    a. None
    b. Iron-folate
    c. Penicillin
    d. Cephalosporins

e. Others (specify)………

27. What medicines were given to take during labour or after you delivered in the hospital?
    a. None
    b. Iron-folate
    c. Penicillin
    d. Cephalosporins
    e. Others (specify)………
28. What medicines have you been given to go with home after delivery?
    a. None
    b. Iron-folate
    c. Penicillin
    d. Cephalosporins
    e. Others (specify)………
29. Who was the healthcare worker who discharged you from the hospital?
    a. Midwife/nurse
    b. Clinical officer
    c. Doctor
    d. Others (specify)…………

A. **Interview Guide:**

1. What are the routine medications given to women during discharge in postnatal ward?
2. What the reasons for giving each of the medicines?
3. What is the practice here regarding giving antibiotics to women at discharge?
4. Which women can get antibiotics during postnatal discharge? (probe for conditions and reasons for giving antibiotics)
5. What are some of the reasons for giving antibiotics to women after a normal delivery?
6. Why do some people give antibiotics for women after normal delivery?
7. What do you think about giving antibiotics to women after a normal delivery?
8. What are some of the benefits of giving antibiotics to women after a normal delivery?
9. What could be the negative consequences of giving antibiotics to women after a normal delivery?
